# Ubiquitin: Not just a one‐way ticket to the proteasome, but a therapeutic dial to fine‐tune the molecular landscape of disease

**DOI:** 10.1002/ctm2.1769

**Published:** 2024-07-17

**Authors:** A. Hyun Kim, P. Murdo Chiknas, Robin E. C. Lee

**Affiliations:** ^1^ Department of Computational and Systems Biology, School of Medicine University of Pittsburgh Pittsburgh Pennsylvania USA

**Keywords:** deubiquitylating enzyme, precision medicine, ubiquitin, ubiquitin modifying enzymes

## Abstract

Recently, there is a rise in studies that recognize the importance of targeting ubiquitin and related molecular machinery in various therapeutic contexts. Here we briefly discuss the history of ubiquitin, its biological roles in protein degradation and beyond, as well as the current state of ubiquitin‐targeting therapeutics across diseases. We conclude that targeting ubiquitin machinery is approaching a renaissance, and tapping its full potential will require embracing a wholistic perspective of ubiquitin's multifaceted roles.

## 1 INTRODUCTION

With every new molecular discovery comes the prospect of exploiting its properties for therapeutic interventions. In 2001, a mutation creating the fusion gene BCR‐ABL1 in chronic myeloid leukaemia (CML) was discovered. The resulting abnormal tyrosine kinase is constitutively active and regulates signals that inhibit apoptosis and promote cell division, leading to a relentless proliferation of cancer cells. Not only did the discovery of BCR‐ABL1 enhance our understanding of leukaemia, but also identified a potent therapeutic target for CML. Imatinib, a small molecule that competes for the active site of BCR‐ABL1 was found to effectively silence the kinase, transforming CML from a life‐threatening condition into a manageable chronic illness for many. The strategy behind Imatinib's success led to sweeping advancements in precision oncology. Now, just over two decades later, over 80 Food and Drug Administration (FDA)‐approved drugs and hundreds more in clinical trials target active sites and kinase‐substrate interactions in inflammatory diseases and cancer.^1^, ^2^

We are once again approaching a moment in translational medicine as pivotal as the introduction of kinase‐targeted cancer therapies: ubiquitin‐modifying enzymes (UMEs) have emerged as powerful candidates for targeted therapy. Numerous studies have started taking a wider view of ubiquitin‐dependent post‐translational modifications (PTMs) and signalling, recognizing their vast clinical potential^3^ (Table 1).  Notably, a recent review article in Clinical and Translational Medicine outlined the regulatory mechanisms of UMEs in cerebrovascular diseases and discussed opportunities for targeted therapy.^4^ Through structural flexibility, dynamic organization, and broad substrate specificity, ubiquitin modifications influence nearly all cellular processes. Here, we briefly discuss the extensive functionality of ubiquitin, emphasizing a more general strategy of targeting ubiquitin machinery in inflammatory diseases and cancer.

**TABLE 1:** A sample of ubiquitin‐modifying enzymes (UMEs) and their targeted therapies. Due to UMEs' direct involvement in cancer, they serve as a ripe target for drug therapies. While a high number of experimental drugs aim to target E3s and deubiquitylases (DUBs), the majority of Food and Drug Administration (FDA)‐approved drugs in this list target the proteasome. This list is not meant to be exhaustive, for an excellent review of therapeutic targets.^35^

Enzyme family	Specific protein (protein family)	Related disease	Targeted drugs
E1 (Ubiquitin activating enzyme)	UBA1	Various cancers	TAK‐243 (MLN7243)^24^
E2 (Ubiquitin‐conjugating enzyme)	UBE2C	Various cancers, including breast and prostate cancer	Investigational drugs^25^
E3 (Ubiquitin ligase)	MDM2 (RING)	Various cancers (due to p53 regulation)	Nutlin‐3^13^ and Idasanutlin^37^
	VHL (Cullin‐RING)	Renal cell carcinoma	PT2385 and PT2977
	BRCA1 (RING)	Breast and ovarian cancer	PARP inhibitors
	Parkin (RBR)	Parkinson's disease	N/A (under investigation)
	CHIP (U‐box)	Neurodegenerative diseases, various cancers and cystic fibrosis	Investigational Drugs^38^, ^39^, ^40^
DUB (Deubiquitinase)	USP7 (HAUSP)	Various cancers	P5091 and P22077
	USP14	Neurodegenerative diseases and multiple myeloma	VLX1570^41^
	CYLD (USP)	Cylindromatosis and various cancers	Subquinocin and Investigational drugs^42^, ^43^
	A20 (OTU)	Autoimmune diseases and lymphomas	Investigational drugs^44^, ^45^
Proteasome	20S/26S Proteasome	Multiple myeloma and mantle cell lymphoma	Bortezomib, Carfilzomib and Ixazomib

### 1.1 Conservation, specificity and dynamics of ubiquitin

Ubiquitin is a small protein composed of 76 amino acids that embodies remarkable sequence and structural similarity across all eukaryotic life, dating back to the last common eukaryotic ancestor. Other studies have shown that a full set of ubiquitin signalling factors, organized in an operon‐like cluster, is present in some archaea and bacteria.^5^ The remarkable conservation of ubiquitin and its associated machinery underscores its evolutionary importance and critical role in biology.

**FIGURE 1:**
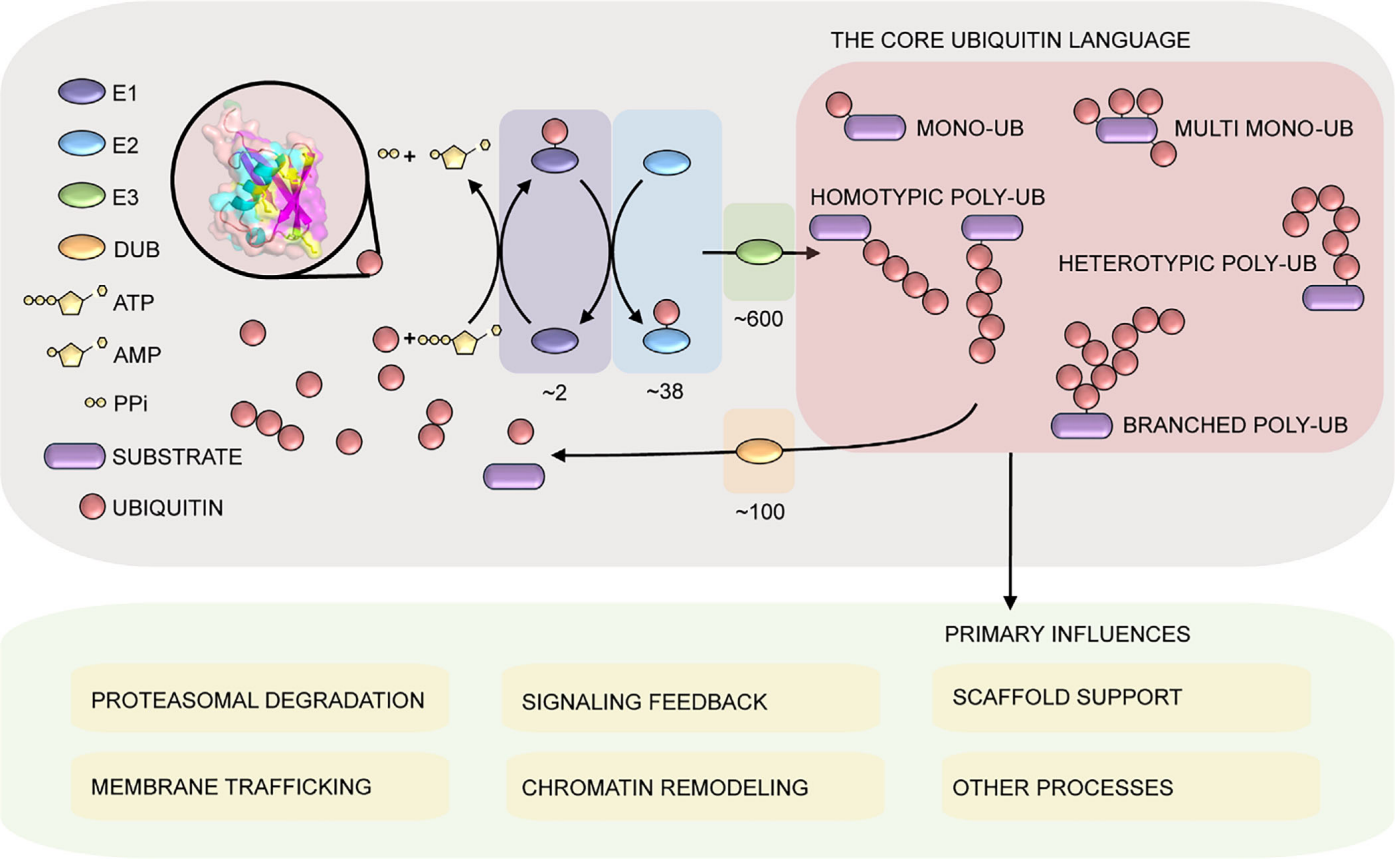
Ubiquitylation by ubiquitin‐modifying enzymes (UMEs): Schematic illustration of the mechanisms of ubiquitin‐modifying enzymes (UMEs) in catalyzing ubiquitylation. E1 ubiquitin‐activating enzymes use ATP to activate ubiquitin. E2 ubiquitin‐conjugating enzymes transfer activated ubiquitin to E3 ligases, which deliver ubiquitin to its substrate. Substrates can be mono‐ubiquitylated (mono‐ub), where a single ubiquitin molecule is attached, or multi‐mono‐ubiquitylated (multi‐mono‐ub), where multiple ubiquitin molecules are attached individually. Alternatively, substrates can be ubiquitylated by a chain of ubiquitin molecules. These chains can be homotypic, sharing a common lysine residue or M1 linkage site, or heterotypic, comprising a combination of different linkage types. Chains can also be branched, influencing the substrate's functionality. The various types of ubiquitylation direct the substrate's cellular function. Ubiquitin monomers and chains can be removed from substrates by deubiquitylases (DUBs), and the released ubiquitin can be recycled by the cell.

Ubiquitylation is a multistep PTM that typically takes place through covalent bonding of the C‐terminal glycine (G76) of a ubiquitin monomer to the lysine side chain of a substrate.^6^ E1 ubiquitin‐activating enzymes initiate the cascade by activating ubiquitin in an ATP‐dependent manner. The activated ubiquitin is transferred to E2 ubiquitin‐conjugating enzymes, which then deliver it to E3 ubiquitin ligases that facilitate its precise attachment to the substrate.^7^ The diversity of UMEs extends beyond E1s, E2s, and E3s to include deubiquitylases (DUBs) that remove ubiquitin from substrates (Figure 1). A notable feature of ubiquitylation is the ability of ubiquitin itself to undergo further ubiquitylation, as illustrated in Figure 1. Each ubiquitin presents eight potential linkage sites (M1, K6, K11, K27, K29, K33, K48, K63),^1^, ^6^ resulting in a vast combinatorial space of possible polyubiquitin chains that encode a large repertoire of biological messages on the targeted substrate (Figure 1).

The interplay between ubiquitylation and deubiquitylation is a highly dynamic push‐pull mechanism that regulates protein turnover and function. The numbers of UMEs vary significantly between the enzyme families, with two E1s, 38 E2s and over 600 E3s, in addition to around 100 DUBs identified in humans.^8^, ^9^ These differences in numbers correspond to varying levels of specificity for each step in ubiquitylation. While the lower numbers of E1s and E2s suggest an overarching mechanism for the activation and creation of ubiquitin codes, the larger numbers of E3s and DUBs reflect tight regulation of substrate targeting and PTM reversal. Think about E3s as “writers” and DUBs as “erasers^6^”: using 600 different coloured pens and 100 different patterned whiteouts, how much detail can we impart onto a single piece of art? Ubiquitylation operates both broadly and with specificity, a molecular toolbox for both the sketches and the masterstrokes, to regulate fundamental cellular processes.

### 1.2 How ubiquitin sculpts the signaling landscape

The discovery of the ubiquitin‐proteasome system (UPS) and its role in protein degradation marked a milestone in cellular biology, culminating in the 2004 Nobel Prize in Chemistry awarded to Ciechanover, Hershko, and Rose. Consequently, the classical textbook view of ubiquitin was minted—ubiquitylation as a PTM that marks substrates for degradation via the UPS. While UPS‐mediated degradation is undoubtedly an essential role of ubiquitin, it does not capture the full breadth of ubiquitin functions. In general, ubiquitylation is equivalent to an information‐dense barcode that provides a host of instructions to substrate proteins, directing their subcellular localization, interaction partners, and activation state among other functions.

Because ubiquitylation is both dynamic and reversible, it provides a control point to fine‐tune signalling dynamics. Ubiquitin‐dependent regulation of p53 exemplifies the principle of dynamic control.^10^ Known for its role in maintaining genomic integrity, the tumour suppressor protein p53 can modulate its own degradation and stabilization through UMEs.^11^, ^12^ Upon activation, p53 induces the expression of MDM2, an E3 ligase that ubiquitylates p53, marking it for proteasomal degradation. Simultaneously, p53 transcriptionally upregulates the DUB enzyme USP7, which counteracts ubiquitylation, thereby stabilizing p53. The opposing regulatory mechanisms finely tune p53 stabilization dynamics, resulting in nuclear p53 and associated gene transcription exhibiting dynamic patterns such as oscillations or tonic accumulation. These different modes of nuclear p53 dynamics regulate DNA damage recovery and cellular commitment to senescence and can be influenced by the cis‐imidazoline analogue Nutlin‐3 that competes with p53 to occupy the MDM2 binding pocket.^13^

Expanding beyond the UPS, ubiquitin can form molecular scaffolds that promote co‐localization of signalling proteins, including those involved in the nuclear factor κB (NF‐κB) pathway—a critical regulator of gene transcription governing immune responses, differentiation, proliferation, and apoptosis. Under basal conditions, NF‐κB molecules are sequestered in the cytoplasm through interactions with inhibitors of NF‐κB (IκB) proteins. However, stimulation by proinflammatory cytokines disrupts IκB‐NF‐κB interactions via phosphorylation of IκB, leading to the release and nuclear translocation of NF‐κB. Essential to NF‐κB control are IκB kinase (IKK) complexes, activated in trans by proximity, typically by forming aggregates with each other and other regulatory kinases. The induced proximity of IKK complexes is facilitated by interaction between their ubiquitin‐binding domains and large polyubiquitin scaffolds assembled near the cytoplasmic tails of ligated receptors. Remarkably, although different inflammatory cytokines converge on similar mechanisms, ubiquitin scaffolds assembled on different cytokine‐receptor complexes rely on distinct ubiquitin chain combinatorics.^14^ Consequently, time‐course dynamics and overall numbers of IKK complexes recruited to ubiquitin scaffolds are cytokine receptor specific. After cytokine exposure, each single cell can produce several hundred distinct scaffolds. Tumour necrosis factor (TNF)‐induced scaffolds can each bring together tens to hundreds of IKK complexes, while IL‐1‐induced scaffolds can each aggregate hundreds of IKK complexes.^15^ Essentially, distinct ubiquitin scaffold requirements encode information within the cell about different extracellular cues, enabling cells to tailor cytokine‐specific responses.

Ubiquitin scaffolds are typically conjugated to specific substrates, with ubiquitin itself sometimes being the substrate. Unanchored polyubiquitin scaffolds originate from several sources, including transcription from genes with three or nine tandem repeats (*UBB* and *UBC* genes, respectively) and en bloc chains removed from other substrates, playing key roles in signalling pathways. For instance, Transforming Growth Factor‐beta‐Activated Kinase 1 (TAK1), which regulates kinases like IKK during inflammatory responses, is directly activated by unanchored K36 polyubiquitin chains.^16^ Similarly, unanchored polyubiquitin can directly activate IKK complexes as well as other signalling pathways to shape responses to immune signals, DNA damage, and environmental stress.^17^

Alongside its roles in receptor‐dependent signalling, ubiquitin also influences membrane trafficking at the plasma membrane. Upon ligand binding, the epidermal growth factor receptor (EGFR) is targeted for internalization into early endosomes via ubiquitylation.^18^ Within these endosomes, sorting complexes recognize ubiquitylated EGFR and sort it into intraluminal vesicles (ILVs) within multivesicular bodies. Lysosomal enzymes subsequently degrade the ubiquitylated cargo in the ILVs. Additionally, DUBs facilitate the recycling of ubiquitin by cleaving it from degraded proteins. In the context of NF‐kB signalling, the removal of ubiquitin from the signalling complex by DUBs following cytokine receptor internalization effectively modulates the duration and intensity of signalling. The resulting ubiquitin monomers are recycled by the cell. This process underscores another aspect of ubiquitin's multifaceted roles, guiding substrates to their proper subcellular destinations while ensuring the continuous recycling of cellular resources crucial for maintaining cellular homeostasis.

Ubiquitin not only shapes protein function but also influences protein synthesis from the outset through its roles in chromatin remodeling.^19^, ^20^ Histones, which are primary units of chromatin structure and epigenetic determinants of gene regulation, undergo various PTMs including ubiquitylation.^21^ The E3 ligase RNF20 mediates ubiquitylation of histone H2B at K120, associated with transcriptional activation, countered by histone DUBs such as USP22. The intersection of histone modifications with ubiquitin‐dependent signalling pathways shapes complex epigenetic programs that profoundly influence gene expression and cell fate.^6^ Ubiquitin's influence spans from quality control of newly synthesized proteins and UPS‐mediated protein degradation to protein trafficking, signalling, and gene expression, playing a pervasive role throughout every stage of a protein's journey.

### 1.3 UMEs’ potential as a therapeutic target

Given ubiquitin's essential roles across cellular processes, the ubiquitin system is a prime target for precision medicine. Despite the diverse regulatory functions of E1s, E2s, E3s, and DUBs, FDA‐approved drugs historically focused on the proteasome. This approach has demonstrated effectiveness; for instance, Bortezomib induces apoptosis in cancer cells by inhibiting the 26S proteasome, preventing the degradation of pro‐apoptotic factors tagged with ubiquitin.^22^ Inhibitors targeting the proteasome and pathways proximal to the UPS have proven successful in treating multiple myeloma and mantle cell lymphomas, though resistance and relapse remain challenges. Second‐generation proteasomes like Carfilzomib and Ixazomib, which similarly target the 20S/26S proteasomal subunits, have enhanced efficacy and ease of use via oral administration with reduced side effects. While valuable, proteasome inhibition targets a fundamental cellular process. Instead, exploring other avenues for precision medicine, such as UMEs and DUBs, holds the potential for overcoming current challenges in treating diverse diseases.

E1s, E2s and E3s offer therapeutic potential with biological implications ranging from nuanced to wide spectrum. Targeting the activation of ubiquitin via E1s has extensive downstream consequences, precluding all processes that require ubiquitin activation. Moreover, a lack of specificity in the mechanism of E1s leads to complex phenotypes that can complicate therapy. An example is TAK‐243, an E1 inhibitor that binds to the tail end of ubiquitin, preventing its activation by an E1 and depleting ubiquitin conjugates in the cell.^23^ Despite a promising start, TAK‐243 encountered substantial clinical challenges due to severe side effects and a restrictive therapeutic window.^24^ Cancer cells often developed resistance, diminishing the feasibility of TAK‐243 for long‐term therapy.

In contrast with E1s, E2s offer a high degree of specificity while still broadly impacting cellular processes by nucleating and extending ubiquitin chains to make “barcodes” that are eventually conjugated to substrate proteins. NSC696723 is an inhibitor of the E2 enzyme Ubc13, which is involved in DNA damage response and immune signalling and has shown significant anti‐tumour activity in preclinical models.^25^ Compared to wide‐spectrum E1 inhibitors, the specificity of NSC697923 for Ubc13 reduces the off‐target effects, but the redundancy and overlapping functions that E2 enzymes mediate are still an issue. Ubc13 has distinct roles in DNA damage tolerance, homologous recombination, telomere resection, inflammatory responses, T cell activation and hypoxia, among many others,^26^ which complicate the development of role‐specific inhibitors.

With over 600 members, E3s are promising targets for precision in drug therapy. As the final arbiters in the ubiquitin‐tagging process, E3 ligases decisively influence the fate of individual substrates, enabling finely tuned interventions with minimal off‐target effects. While advantageous, high therapeutic specificity demands precise targeting of individual E3 ligases, which can vary between cell types and disease states. Additionally, the intricate network of E3 ligases is a complex system that, like many biological networks, has not been fully mapped, and successful targeting may still lead to unintended cellular responses. Thalidomide, originally intended to treat morning sickness during pregnancy, binds to the E3 component cereblon (CRBN) and alters its substrate specificity, resulting in the ubiquitylation and degradation of proteins not normally targeted by CRBN. These off‐target proteins include those involved in fibroblast growth factor signalling, leading to teratogenic effects that cause severe limb malformation in affected embryos.^27^ Conversely, modern approaches such as proteolysis targeting chimaeras (PROTACs) underscore the advantages of E3‐targeting drugs. PROTACs enhance protein degradation by catalyzing E3 activity through increased proximity between ligase and substrate.^28^ ARV‐110, a PROTAC targeting the androgen receptor, has shown notable promise in precise treatment for prostate cancer with minimized off‐target effects.^29^

There is a growing array of E3‐targeting small molecules, many using steric hindrance to selectively disrupt enzyme‐substrate interactions, exemplified by Nutlin‐3 occupying MDM2's substrate interaction pocket, as described above. Such ‘edgetic’ approaches^30^ target specific protein‐protein interactions (PPIs) between E3s and their substrates without compromising overall E3 or ubiquitin system function. For example, using computational screening and molecular docking, Pabon et al. identified small molecules disrupting TNF‐induced polyubiquitin scaffolds.^31^ Employing a ‘network‐centric’ approach, they pinpointed two small molecules that specifically block the recruitment of TRAF2 to activated TNF‐receptor complexes, selectively inhibiting TNF‐induced NF‐kB signaling while preserving other cytokine‐induced polyubiquitin scaffolds. Similarly, inh‐2 is a small molecule that selectively targets the E3 ligase RNF5, known for its role in degrading misfolded CFTR proteins.^32^ Upon binding, inh‐2 stabilizes CFTR, preventing RNF5 from recognizing and ubiquitylating it, and has shown promising anti‐tumor activity in preclinical studies by inhibiting tumor growth and promoting apoptosis in various cancer models. Smac mimetics, which mimic the function of Smac/DIABLO,^33^ are another class of small molecules that induce apoptosis by inhibiting E3 ligase inhibitor of apoptosis proteins (IAPs), demonstrating clinical promise in treating various cancers.^34^ These are only a few examples; in a recent issue of Clinical and Translational Medicine, Sampson et al. provide an excellent review of E3‐targeting therapeutics in cancer.^35^

Building on the advancement of E3‐targeting drugs, significant progress is also being made in targeting DUBs. One such example demonstrating potent anti‐tumour activity in preclinical models is P5091, an inhibitor of DUB enzyme USP7, which is involved in regulating the tumour suppressor p53. The specificity of P5091 for USP7 reduces off‐target effects compared to inhibitors with widespread biological impact. However, a challenge persists due to the redundancy and overlapping functions of DUBs. USP7 plays several roles in DNA damage response, cell cycle regulation, apoptosis, and others, complicating the development of specific inhibitors. Additionally, like many chemical therapeutics, DUB inhibitors may induce resistance mechanisms that limit their therapeutic efficacy.

Much like kinases, UMEs are crucial regulators of commonly disrupted biological processes in disease, with great potential as targets for precision medicine. Compared to protein kinases, there are even greater numbers of potential molecular targets between E1s, E2s, E3s, and DUBs, with the ability to target UMEs with broad spectrum or highly selective enzymatic and PPI‐disrupting reactivities. In parallel with ongoing advancements in kinase research, continued research into UMEs will be pivotal for developing effective single‐entity and combination therapies across diverse diseases.

## 2 CONCLUSION

Initially discovered in the context of regulated protein degradation, our scientific understanding of ubiquitin has evolved to recognize the panoply of its fundamental biological roles. In just a few decades, our comprehension of ubiquitin's regulatory potential has expanded enormously. As we prepared this commentary, we were reminded of lessons from Yuri Lazebnik's humorous and deeply insightful correspondence, “Can a biologist fix a radio?—Or, what I learned while studying apoptosis^36^”. Two lessons in particular stand out in the context of ubiquitin. First, we must occasionally be reminded of the biological discovery process: the more we learn about seemingly simple processes, the less we seem to understand—until we step back to synthesize and reconcile our growing knowledge with the thinking and terminology used early in the discovery phase. Second, we should approach biological discoveries formally as complex systems.

Regarding the former, the abbreviation of ‘Really Interesting New Gene’ to RING‐finger domain E3s serves as an iconic and almost ironic example. Yet, the name ‘ubiquitin’ itself is apt, symbolizing a protein conserved widely across eukaryotes and crucial to most biological processes—ubiquitin can be a ticket to the proteasome, but that is just one message in a complex language that ubiquitin mediates. As for the latter, we have only scratched the surface and are moving towards systems approaches that map the interaction landscape, promising to reveal the entirety of the complex language of ubiquitin. With increasing knowledge and the discovery of new interventions, there is enormous potential to revolutionize precision therapies targeting the molecular underpinnings of ubiquitin across diverse diseases—from immunity to cerebrovascular diseases and beyond.

## AUTHOR CONTRIBUTIONS

Conceptualization: A. Hyun Kim, P. Murdo, Chiknas and Robin E. C. Lee; Writing: A. Hyun Kim, P. Murdo Chiknas and Robin E. C. Lee; Supervision and Funding Acquisition: Robin E. C. Lee

## ETHICS STATEMENT

The manuscript does not involve any research with human or animal subjects, and ethical approval is not applicable.

## References

[1] French ME , Koehler CF , Hunter T . Emerging functions of branched ubiquitin chains. Cell Discov. 2021;7(1):6. 33495455 10.1038/s41421-020-00237-y PMC7835216

[2] Lu H , Zhou Q , He J , et al. Recent advances in the development of protein‐protein interactions modulators: mechanisms and clinical trials. Signal Transduct Target Ther. 2020;5(1):213. 32968059 10.1038/s41392-020-00315-3 PMC7511340

[3] Turnbull EL , Rosser MFN , Cyr DM . The role of the UPS in cystic fibrosis. BMC Biochem. 2007(1):S11. 8 Suppl 1. Suppl. 18047735 10.1186/1471-2091-8-S1-S11 PMC2106362

[4] Huang J , Zhu Z , Schlüter D , Lambertsen KL , Song W , Wang Xu . Ubiquitous regulation of cerebrovascular diseases by ubiquitin‐modifying enzymes. Clin Transl Med, 2024;14(5):e1719. 10.1002/ctm2.1719 PMC11111633 38778460

[5] Zuin A , Isasa M , Crosas B . Ubiquitin signaling: extreme conservation as a source of diversity. Cells. 2014;3(3):690‐701. 25014160 10.3390/cells3030690 PMC4197634

[6] Oh E , Akopian D , Rape M . Principles of ubiquitin‐dependent signaling. Annu Rev Cell Dev Biol. 2008;34:137‐162. 10.1146/annurev-cellbio-100617-062802 30110556

[7] Scheffner M , Nuber U , Huibregtse JM . Protein ubiquitination involving an E1–E2–E3 enzyme ubiquitin thioester cascade. Nature. 1995;373:81‐83. 7800044 10.1038/373081a0

[8] Ye Y , Rape M . Building ubiquitin chains: E2 enzymes at work. Nat Rev Mol Cell Biol. 2009;10(11):755‐764. 19851334 10.1038/nrm2780 PMC3107738

[9] Rape M . Ubiquitylation at the crossroads of development and disease. Nat Rev Mol Cell Biol. 2018;19(1):59‐70. 28928488 10.1038/nrm.2017.83

[10] Park J‐H , Zhuang J , Li J , Hwang PM . p53 as guardian of the mitochondrial genome. FEBS Lett. 2016;590(7):924‐934. 26780878 10.1002/1873-3468.12061 PMC4833664

[11] Purvis JE , Lahav G . Encoding and decoding cellular information through signaling dynamics. Cell. 2013;152(5):945‐956. 23452846 10.1016/j.cell.2013.02.005 PMC3707615

[12] Nag S , Qin J, Srivenugopal KS, Wang M, Zhang R. The MDM2‐p53 pathway revisited. J Biomed Res. 2013;27(4):254‐271. 23885265 10.7555/JBR.27.20130030 PMC3721034

[13] Purvis JE , Karhohs KW , Mock C , Batchelor E , Loewer A , Lahav G . p53 dynamics control cell fate. Science. 2012;336(6087):1440‐1444. 22700930 10.1126/science.1218351 PMC4162876

[14] Tarantino N , Tinevez J‐Y , Crowell EF , et al. TNF and IL‐1 exhibit distinct ubiquitin requirements for inducing NEMO–IKK supramolecular structures. J Cell Biol. 2014;204(2):231‐245. 24446482 10.1083/jcb.201307172 PMC3897181

[15] Cruz JA , Mokashi CS , Kowalczyk GJ , et al. A variable‐gain stochastic pooling motif mediates information transfer from receptor assemblies into NF‐kB. Sci Adv. 2021;7(30). 10.1126/sciadv.abi9410 PMC8302133 34301608

[16] Xia Z‐P , Sun L , Chen X , et al. Direct activation of protein kinases by unanchored polyubiquitin chains. Nature. 2009;461(7260):114‐119. 19675569 10.1038/nature08247 PMC2747300

[17] Blount JR , Johnson SL , Todi SV . Unanchored ubiquitin chains, revisited. Front Cell Dev Biol. 2020;8:582361. 33195227 10.3389/fcell.2020.582361 PMC7659471

[18] Schuh AL , Audhya A . The ESCRT machinery: from the plasma membrane to endosomes and back again. Crit Rev Biochem Mol Biol. 2014;49(3):242‐261. 24456136 10.3109/10409238.2014.881777 PMC4381963

[19] Guo P , Hoang N , Sanchez J , et al. The assembly of mammalian SWI/SNF chromatin remodeling complexes is regulated by lysine‐methylation dependent proteolysis. Nat Commun. 2022;13(1):6696. 36335117 10.1038/s41467-022-34348-9 PMC9637158

[20] Challa K , Schmid CD , Kitagawa S , et al. Damage‐induced chromatome dynamics link Ubiquitin ligase and proteasome recruitment to histone loss and efficient DNA repair. Mol Cell. 2021;81(4):811‐829. e6. 33529595 10.1016/j.molcel.2020.12.021

[21] Ulrich HD , Walden H . Ubiquitin signalling in DNA replication and repair. Nat Rev Mol Cell Biol. 2010;11(7):479‐489. 20551964 10.1038/nrm2921

[22] Weathington NM , Mallampalli RK . Emerging therapies targeting the ubiquitin proteasome system in cancer. J Clin Invest. 2014;124(1):6‐12. 24382383 10.1172/JCI71602 PMC3871250

[23] Hyer ML , Milhollen MA , Ciavarri J , et al. A small‐molecule inhibitor of the ubiquitin activating enzyme for cancer treatment. Nat Med. 2018;24(2):186‐193. 29334375 10.1038/nm.4474

[24] Sherman DJ , Li J . Proteasome Inhibitors: harnessing proteostasis to combat disease. Molecules. 2020;25(3):671. 10.3390/molecules25030671 PMC7037493 32033280

[25] Hodge CD , Edwards RA , Markin CJ , et al. Covalent inhibition of Ubc13 affects ubiquitin signaling and reveals active site elements important for targeting. ACS Chem Biol. 2015;10(7):1718‐1728. 25909880 10.1021/acschembio.5b00222 PMC4506735

[26] Curtis D , Hodge LS , Glover JNM . Ubc13: the Lys63 ubiquitin chain building machine. Oncotarget. 2016;7(39):64471‐64504. 10.18632/oncotarget.10948 PMC5325457 27486774

[27] Rehman W , Arfons LM , Lazarus HM . The rise, fall and subsequent triumph of thalidomide: lessons learned in drug development. Ther Adv Hematol. 2011;2(5):291‐308. 23556097 10.1177/2040620711413165 PMC3573415

[28] Sakamoto KM , Kim KB , Kumagai A , Mercurio F , Crews CM , Deshaies RJ . Protacs: chimeric molecules that target proteins to the Skp1‐Cullin‐F box complex for ubiquitination and degradation. Proc Natl Acad Sci U S A. 2001;98(15):8554‐8559. 11438690 10.1073/pnas.141230798 PMC37474

[29] Li X , Song Y . Proteolysis‐targeting chimera (PROTAC) for targeted protein degradation and cancer therapy. J Hematol Oncol. 2020;13(1):50. 32404196 10.1186/s13045-020-00885-3 PMC7218526

[30] Sahni N , Yi S , Zhong Q , et al. Edgotype: a fundamental link between genotype and phenotype. Curr Opin Genet Dev. 2013;23(6):649‐657. 24287335 10.1016/j.gde.2013.11.002 PMC3902775

[31] Pabon NA , Zhang Q , Cruz JA , Schipper DL , Camacho CJ , Lee REC . A network‐centric approach to drugging TNF‐induced NF‐kappaB signaling. Nat Commun. 2019;10(1):860. 30808860 10.1038/s41467-019-08802-0 PMC6391473

[32] Sondo E , Falchi F , Caci E , et al. Pharmacological inhibition of the ubiquitin ligase RNF5 rescues F508del‐CFTR in cystic fibrosis airway epithelia. Cell Chem Biol. 2018;25(7):891‐905. e8. 29754957 10.1016/j.chembiol.2018.04.010

[33] Martinez‐Ruiz G , Maldonado V , Ceballos‐Cancino G , Grajeda JPR , Melendez‐Zajgla J . Role of Smac/DIABLO in cancer progression. J Exp Clin Cancer Res. 2008;27(1):48. 18822137 10.1186/1756-9966-27-48 PMC2566557

[34] Bai L , Smith DC , Wang S . Small‐molecule SMAC mimetics as new cancer therapeutics. Pharmacol Ther. 2014;144(1):82‐95. 24841289 10.1016/j.pharmthera.2014.05.007 PMC4247261

[35] Sampson C , Wang Q , Otkur W , et al. The roles of E3 ubiquitin ligases in cancer progression and targeted therapy. Clin Transl Med. 2023;13(3):e1204. 36881608 10.1002/ctm2.1204 PMC9991012

[36] Lazebnik Y . Can a biologist fix a radio?–Or, what I learned while studying apoptosis. Cancer cell. 2002;2(3):179‐182. 12242150 10.1016/s1535-6108(02)00133-2

[37] Khurana A , Shafer DA . MDM2 antagonists as a novel treatment option for acute myeloid leukemia: perspectives on the therapeutic potential of idasanutlin (RG7388). Onco Targets Ther. 2019;12:2903‐2910. 31289443 10.2147/OTT.S172315 PMC6563714

[38] Liu C , Lou W , Yang JC , et al. Proteostasis by STUB1/HSP70 complex controls sensitivity to androgen receptor targeted therapy in advanced prostate cancer. Nat Commun. 2018;9(1):4700. 30446660 10.1038/s41467-018-07178-x PMC6240084

[39] Liu Y , Zhou H , Tang X . STUB1/CHIP: new insights in cancer and immunity. Biomed Pharmacother. 2023;165:115190. 37506582 10.1016/j.biopha.2023.115190

[40] Xu P , Yang JC , Ning S , et al. Allosteric inhibition of HSP70 in collaboration with STUB1 augments enzalutamide efficacy in antiandrogen resistant prostate tumor and patient‐derived models. Pharmacol Res. 2023;189:106692. 36773708 10.1016/j.phrs.2023.106692 PMC10162009

[41] Wang X , Mazurkiewicz M , Hillert E‐K , et al. The proteasome deubiquitinase inhibitor VLX1570 shows selectivity for ubiquitin‐specific protease‐14 and induces apoptosis of multiple myeloma cells. Sci Rep. 2016;6:26979. 27264969 10.1038/srep26979 PMC4893612

[42] Huang Z , Tan Y . The potential of cylindromatosis (CYLD) as a therapeutic target in oxidative stress‐associated pathologies: a comprehensive evaluation. Int J Mol Sci. 2023;24(9):8368. 10.3390/ijms24098368 PMC10179184 37176077

[43] Yamanaka S , Sato Y , Oikawa D , et al. Subquinocin, a small molecule inhibitor of CYLD and USP‐family deubiquitinating enzymes, promotes NF‐kappaB signaling. Biochem Biophys Res Commun. 2020;524(1):1‐7. 31898971 10.1016/j.bbrc.2019.12.049

[44] Catrysse L , Vereecke L , Beyaert R , Van Loo G . A20 in inflammation and autoimmunity. Trends Immunol. 2013;35(1):22‐31. 24246475 10.1016/j.it.2013.10.005

[45] Momtazi G , Lambrecht BN , Naranjo JR , Schock BC . Regulators of A20 (TNFAIP3): new drug‐able targets in inflammation. Am J Physiol Lung Cell Mol Physiol. 2019;316(3):L456‐L469. 30543305 10.1152/ajplung.00335.2018

